# Refusal of Emergency Medical Transport After a Fall: Patient Characteristics and Outcomes of Repeat Callers

**DOI:** 10.5811/westjem.33524

**Published:** 2025-08-20

**Authors:** Jacob Barr, Katherine Selman, Krystal Hunter, Alexander Kuc

**Affiliations:** * Cooper Medical School of Rowan University, Camden, New Jersey; † Cooper University Health Care, Department of Emergency Medicine, Camden, New Jersey

## Abstract

**Introduction:**

Lift assistance represents a high proportion of emergency medical services (EMS) calls, yet data is limited regarding the long-term outcomes of these patients who subsequently refuse transport. In this study, our objective was to examine the outcomes of patients who require lift assistance but refuse transport and to determine factors associated with repeated EMS utilization.

**Methods:**

We conducted a retrospective, observational cohort study of EMS calls in southern New Jersey in patients ≥18 years of age who declined EMS transport after a fall between July 1, 2021–July 1, 2022. Repeat callers were defined as making one additional call within 30 days, and we defined super-users as those making ≥four calls within six months. The primary outcome was repeat emergency department (ED) visits within 30 days from the initial transport refusal visit.

**Results:**

We analyzed 116 of 203 (57%) patients. The mean patient age was 66.3 years, and 53.6% were female. Forty-seven (37.9%) patients were repeat callers, and 27 (21.8%) were super-users. Repeat callers and super-users had increased odds of 30-day ED visits (odds ratio [OR] 17.2, 95% confidence interval [CI] 6.4–47.6, and OR 8.8, 95% CI 3.3–23.7, respectively), and six-month ED visits (OR 4.9, 95% CI 2.2–11.2, and OR 12.9, 95% CI 3.9–56.5). Similarly, there were increased odds of 30-day admission for repeat and super-user callers (OR 6.6, 95% CI 2.5–18.2, and OR 10.8, 95% CI 4.0–29.8, respectively), and six-month admissions (OR 3.0, 95% CI 1.4–6.5,and OR 6.8, 95% CI 2.6–19.9, respectively). No differences in death at one year were observed in either group (repeat callers OR 1.4, 95% CI 0.4–4.5; super-users OR 1.1, 95% CI 0.2–4.1) Repeat callers had higher proportions of anticoagulation/antiplatelet therapy and non-ambulatory status (42.9% vs 61.7%, *P*=.046 and 29.0% vs 56.8%, *P*=.006, respectively).

**Conclusion:**

Repeat EMS calls for lift assistance may be used to identify patients at high risk for ED visits and hospitalizations. As patients decline transport, EMS may be their sole healthcare encounter. Future directions would entail training EMS personnel in screening or referring patients for more intensive outpatient interventions.

## INTRODUCTION

Older adults experience an estimated 36 million falls yearly in the United States, with 28% of adults >65 of age reporting falls in 2020.^1^,^2^ Falls account for nearly 2% of emergency medical services (EMS) calls, but rises to 11.5% in adults >60.^3^,^4^ Falls are a national problem, with fall-related EMS calls increasing by 268% from 2007 to 2017.^4^,^5^ Further concerning, while 5.1% of all EMS calls for adults result in refusal of care following EMS contact, fall-victim refusal rates range from 11–56%.^6^,^7^ Within this study population, 49% of fall victims had an unplanned healthcare encounter within 28 days.^7^ This suggests that lift assists, or assistance after a fall without intent for EMS transport, may represent a sentinel event for patient care. Previous studies demonstrated that repeated calls for lift assists represent over a quarter of the study population, and half of lift-assist patients required that EMS return to the same address within 30 days, representing a large portion of EMS and paramedic resource utilization.^3^,^8^,^9^

While data exists that analyzes demographic, medical history, and social factors for those who refuse EMS transportation, limited data analyzes short- and long-term healthcare utilization of fall victims who refuse EMS assistance following their initial lift-assist encounter. Our objective was to describe the prevalence of repeat calls among patients who suffered falls or required lift assistance but declined initial EMS transport, to identify demographic information and factors that may contribute to increased EMS utilization, and to assess 30-day and six-month ED visits and hospitalizations.

## METHODS

### Study Design and Population

We conducted a retrospective cohort study of “fall victims” who refused transport in Camden County, New Jersey, treated by Cooper University Health Care (CUHC) EMS between July 1, 2021_July 1, 2022. Cooper EMS’ structure and volume are described in Supplement 1. We followed distinct criteria posed by Worster et al (Supplement 2).^10^

We identified patients were identified using the Zoll emsCharts system (Zoll Medical, Chelmsford, MA) to track community-dwelling patients ≥18 years of age classified as “fall victims” who then refused medical transport to the hospital following evaluation (Broomfield, CO, Supplement 2). Exclusion criteria included patients <18 years of age and those residing in assisted living, skilled nursing, long-term care, or memory care facilities. These individuals were excluded as they rarely refuse transport or may have access to appropriate medical resources (primary care, ancillary staff, etc) unavailable to community members.

The hospital electronic health record (EHR) (Epic Systems Corporation, Verona, WI) was assessed for our independent and outcome variables. Emergency medical services transport patients to CUHC, Virtua Camden, or Our Lady of Lourdes Hospital in Camden, NJ, all of which have Epic as their primary EHR. If patients were transported to hospital systems using different EHRs, their outcome variables would not be accessible. Demographic information and additional characteristics collected are described in Supplement 2.

High utilization in the emergency setting is defined variably in the literature. 11 The supplemental material describes our rationale for defining both repeat callers and super-users. Repeat callers were patients who required EMS services at least one additional time within 30 days following their fall.^12^ Super-user patients were defined as patients with ≥four calls in six months.

**Population Health Research Capsule**

What do we already know about this issue?

*Fall-victim refusal of EMS transport ranges from 11–56%, and half of fall victims have an unplanned healthcare encounter within 28 days*.

What was the research question?

*Assess ED visits and hospitalizations among patient who suffered falls, but declined initial EMS transport*.

What was the major finding of the study?

*30-day ED visits were increased in repeat callers (OR 17.2, 95% CI 6.4–47.6) and super-users (OR 8.8, 95% CI 3.3–23.7)*.

How does this improve population health?

*EMS responders may be the only medical personnel to be aware of patient falls. The approach to fall victims should be structured to address short- and long-term outcomes*.

### Outcomes

Our primary outcome was the odds of ED visits within 30 days from the initial transport refusal. Secondary outcomes included odds of ED visits in six months, hospital admission at 30 days and six months, and death. Data analysis methods can be found in the supplemental materials. This project received institutional review board approval from CUHC (IRB Number 22–245).

## RESULTS

### Demographics and Call Characteristics

Between July 1, 2021–July 1, 2022, 203 unique calls were made to EMS for “fall victims” who subsequently declined transport, and 194 fit our inclusion criteria. Of those calls, 18 patients accounted for 63 EMS encounters, while the remaining 131 patients accounted for only one encounter during the study period. Twenty-five patients did not have identifying information documented by EMS, and eight patients did not have a medical record number to connect their EMS and EHR documentation. This left 116 individuals for analysis (Figure S1). The initial call was chosen as the primary analysis point for patients with multiple EMS dispatches.

Of the 149 unique patients, 55% were Black and 53.6% were female, with a mean age of 66.3 years of age (Table 1). When comparing demographic information of repeat and non-repeat callers, repeat callers had higher rates of taking anticoagulation or antiplatelet medications (42.9% vs 61.7%, *P* = .05) and being non-ambulatory (29.0% vs 56.8%, *P* =.001) (Table S1).

### ED visits and Hospital Admissions of Repeat Callers and Super-users

Forty-seven (37.9%) patients were repeat callers, and 27 (21.8%) were super-users. The odds ratio (OR) of ED visits in 30 days for repeat callers compared to non-repeat callers was 17.2 (95% confidence interval [CI] 6.4–47.6) (Table 2). For 6-month ED visits, this relationship also held (OR 4.9, 95% CI 2.2–11.2). Repeat callers had increased odds of admission both at 30 days (OR 6.6, 95% CI 2.5–18.2) and six months (OR 3.0, 95% CI 1.4–6.5). There was no difference in one-year mortality. Super-users demonstrated no significant difference in demographics or vital signs from non-super-users (Table S1). Super-users had increased odds of ED visits within 30 days and six months (OR 8.8, 95% CI 3.3–23.7, and OR 12.9, 95% CI 3.9–56.5, respectively) (Table 2). Similarly, there were also increased odds of hospitalizations at 30 days and six months (OR 10.8, 95% CI 4.0–29.8, and OR 6.8, 95% CI 2.6–19.9, respectively). There was no difference found between anticoagulation or antiplatelet status in these patients (*P*=.543, Table S1). No difference in one-year mortality between the super-users and non-super-users was found.

## DISCUSSION

In the present study, we demonstrate an increased odds of 30-day ED visits and hospitalization and six-month ED visits and hospitalization for both repeat EMS callers and EMS super-users who decline transport after a fall. Our study is different from prior studies in several ways. Our patient population was 9–12 years younger than patients in prior studies, meaning EMS personnel should be aware that a younger cohort of patients is still at risk of needing medical assistance in the immediate future.^3^,^13^,^14^ Further, our study stratifies patients into either repeat callers or super-users of EMS to assess 30-day and six-month outcomes, while others looked at 14-day outcomes for lift-assist patients and did not further analyze this distinct subset of patients.^3^,^15^ Finally, Mikolaizak et al demonstrated that 49% of fall victims experienced unplanned healthcare assessments within 28 days.^7^ Our study shows a higher percentage of 30-day ED visits for both repeat callers and super-users (66% and 70.4%, respectively, Table 2). This suggests that the prehospital approach to fall victims should be structured to address short- and long-term outcomes.

Anti-coagulation and ambulatory status in repeat callers, but not super-users, was the only statistically significant medical history difference between repeat callers. Thus, the presence or absence of certain demographic or medical characteristics is unlikely to be generalizable, and we may need to consider other factors when triaging fall victim patients, including the fall location (public vs private space), fall-related EMS call history, depression, polypharmacy, and diabetes.^16^,^17^,^18^

The EMS responders may be the first or only medical personnel to be aware of patient falls, as patients may under-report falls to their primary care practitioners.^19^ One study has demonstrated EMS positively screened 61% of future fall risk.^20^ However, screening and education are tools for identifying at-risk patients but may not address risk factors for future falls.^21^ Emergency medical services referral programs also have demonstrated moderate success with patient referral for social service-related events.^22^

## LIMITATIONS

This was a retrospective, observational study reliant on chart review. We were unable to determine living arrangements or ambulation status in almost one-third of patients, factors that may only be documented if the EMS responder thought it was relevant. Cognitive impairment and other potentially relevant characteristics may not have been charted by EMS. This limits conclusions for which patient characteristics are highly associated with increased EMS use and at risk for future healthcare utilization. Additionally, only calls classified as “fall victims” in the prehospital EHR were assessed. Because the calls are characterized by the EMS dispatchers, patients may have been misclassified. We may have also missed patients if they used a different EMS company for repeat calls and were transferred to hospitals outside of the CUHC EMS catchment area.

Additionally, we did not find a significant difference in patients with abnormal or incomplete vital signs. In instances where vital signs were abnormal, EMS may have been able to convince patients to go to the ED. This would represent patients who initially wanted to decline transport, but EMS intervened to persuade these higher risk patients to seek treatment.

## CONCLUSION

Anticoagulation and non-ambulatory status are associated with patients who use EMS after a fall but subsequently decline medical transport. Patients with repeated EMS calls are significantly more likely to have ED visits and hospital admissions at 30 days and six months after the initial fall.

## Supplementary Information



## Figures and Tables

**Table 1:** Characteristics of patient calls.

Characteristic	All study subjects (N=149)
Sex *n* (%)	
Female	80 (53.6)
Male	62 (41.6)
Unknown/Missing	7 (4.7)
Race *n* (%)	
White	32 (21.4)
Black	82 (55.0)
Asian	0
American Indian	0
Other	35 (23.5)
Unknown/missing	0
Ethnicity	
Hispanic	26 (17.4)
Non-Hispanic	98 (65.8)
Unknown	25 (16.8)
Age, years (*mean ± SD*)	66.3 ± 16.7
Service Used *n* (%)	
BLS	146 (98.0)
ALS	3 (2.0)
Dementia *n* (%)	
Present	7 (4.7)
Absent	114 (76.5)
Unknown	28 (18.8)
Altered mental status *n* (%)	
Present	3 (2.0)
Absent	136 (91.3)
Unknown	10 (6.7)
Anticoagulation *n* (%)	
Present	59 (39.6)
Absent	61 (40.9)
Unknown	29 (19.5)
Completed vital signs *n* (%)	
Yes	88 (59.1)
No	61 (40.9)
Living conditions *n* (%)	
Alone	56 (37.6)
With others	50 (33.5)
Unknown	43 (28.9)
Ambulatory status *n* (%)	
Walking	72 (48.3)
Wheelchair/ bedbound	45 (30.2)
Unknown	32 (21.5)

*BLS*, Basic Life Support; *ALS*, Advanced Life Support.

**Table 2:** Proportion of repeat/non-repeat callers and super utilizers/non-super users and their odds ratio for specific emergency department visits and hospital admissions in 30 days/six months and deaths.

	Non-repeat (n = 69)	Repeat (n=47)	Odds ratio (95% CI)
ED Visits in 30 days, n (%)	7(10.1)	31 (66.0)	17.2 (6.4–47.6)
ED Visits in 6 months, n (%)	7(10.1)	20 (42.6)	4.9 (2.2–11.2)
Admission in 30 days, n (%)	24 (34.8)	34 (72.3)	6.6 (2.5–18.2)
Admission in 6 months, n (%)	20 (28.9)	26(47)	3.0 (1.4–6.5)
Death at one year, n (%)	7 (10.1)	6 (12.2)	1.4 (0.4–4.5)
	Non-super-user (n = 89)	Super-user (n = 27)	Odds ratio (95% CI)
ED Visits in 30 days n (%)	19 (21.3)	19 (70.4)	8.8 (3.3–23.7)
ED Visits in 6 months n (%)	16 (18.0)	19 (70.4)	12.9 (3.9–56.5)
Admissions in 30 days n (%)	34 (38.2)	24 (88.9)	10.8 (4.0–29.8)
Admissions in 6 months n (%)	26 (29.2)	20 (74.1)	6.8 (2.6–19.9)
Deaths n (%)	10 (11.2)	3 (11.1)	1.1 (0.2–4.1)

*ED*, emergency department; *CI*, confidence interval.
