# Supplementary material for: Refusal of Emergency Medical Transport After a Fall: Patient Characteristics and Outcomes of Repeat Callers
Source: West J Emerg Med. 2025 Aug 20;26(5):1291–5. doi: 10.5811/westjem.33524 (PMC12591631; doi:10.5811/westjem.33524)
Supplement: Supplementary file 1 [file wjem-26-1291-s001.docx]

**Supplemental Materials**

**Supplemental 1.** Description of Cooper University Health Care(CUHC) EMS Services: CUHC EMS is a hospital-based, two-tiered 9-1-1 service in a primarily urban setting utilizing basic life support (BLS) units staffed by EMTs and advanced life support (ALS) units staffed by paramedics, with additional support from a 24-hour paramedic supervisor and EMS physician response units. The annual volume of 9-1-1 calls was approximately 70,000 during the study period. CUHC EMS transfers patients to either Cooper University Health Care or Virtua Our Lady of Lourdes in Camden, NJ. If a patient fell in Camden, NJ and was transferred to either of those hospitals, we should have access to both their Zoll emsCharts documentation and Epic EHR documentation. However, if that same patient utilized EMS outside of CUHC and was transferred to a different hospital, then we would not have access to their hospital course information.

**Supplemental 2.** Additional Methods

- Worster et. al recommendations we followed: abstractor training, case selection criteria, variable definition, abstraction forms, performance monitoring, medical record identification, sampling method, missing-data management plan, and institutional review board approval.^1^ The abstractors were not blind to the hypothesis and interobserver reliability was not measured as author JB was the sole data abstractor, who knew the study hypothesis prior to data collection.
- Fall Victim Criteria in Zoll emsCharts: To assess patients not transported to the hospital, search criteria included any patient documented as “lift assist,” “patient refusal by action,” “patient refused care,” “patient refusal,” “refused: billed,” “refused: not billed,” “treated and released,” or “treated, refused against medical advice”. These outcomes are inputted by EMS following the call’s completion and represent a comprehensive list of possible outcomes if patient refuse care.
- Demographic information included the patients’ self-reported age, sex, and race. Additional characteristics of each patient recorded included type of transport (BLS or ALS), documented history of dementia, documented altered mental status, anticoagulation or antiplatelet medication status, presence of completed vital signs, living status (whether the patient lives independently or with others), and documented ambulatory status (defined as ambulatory, including use of cane or walker, or non-ambulatory, which included patients who utilize wheelchairs or are confined to bed). A complete set of vital signs, as defined by our EMS physicians, includes heart rate, blood pressure, oxygen saturation, respiratory rate, and Glascow Coma Score (GCS). Patients with a documented history of dementia or cognitive impairment were deemed to have altered mental status if their charts indicated deviations from baseline, per collateral from care partners. Anticoagulation or antiplatelet status was determined by patient medication lists at the time of the initial encounter. Household occupants and ambulatory status were recorded if specified in their charts or the narrative of the EMS providers. Each patient’s medical record number (MRN) was only used one time to ensure multiple patients were not included in the subset of the study population.
- Rationale for Repeat Caller Definition: Based on prior work showing 1 in 6 older adults had repeat EMS transport within 30 days with an association of falls with repeat transports. ^2^
- Rationale for Super-Utilizer Definition: Super Utilizers have been previously qualified variably as well with thresholds of anywhere between 3 and 10 ED visits per year, 3 to 10 visits per 6 month period, or based on patient percentile. ^3,4^ Frequent utilization has been less explored in EMS literature but have been described as high-frequent utilization for 7 to 10 transports per year and super-frequent utilization as 11 or more transports per year. ^5^
- Data analysis: Age, heart rate, blood pressure, oxygen saturation, and respiratory rate were compared using independent t-tests. Chi-square testing was used to compare the remainder of variables including race, sex, ALS or BLS unit utilization, history of dementia, anti-coagulation status, presence of altered mental status, complete vital signs, and ambulatory status. The odds of ED visits in 30 days and six months, hospital admissions in 30 days and six months, and death were calculated using the non-repeat and super-utilizer statistics as the basis for comparison. A p-value of <0.05 was considered statistically significant. When determining statistical relationships between demographics and EMS calls, percentages represent the proportions of known outcomes and exclude patients who did not have adequate outcome measures in the EHR. Analysis was completed using SPSS (IBM, Armonk, NY).

**Tables and Figures**

**Figure S1.** CONSORT Diagram used when analyzing primary patient outcomes

**Table S1.** Demographics, Call Characteristics, and Vital Signs Comparison of Repeat (≥1 additional Call in 30 Days) vs Non-Repeat Patients and Super Utilizers ( ≥ 4 calls in 6 months) vs Non-Super Utilizers

|  | Non-Repeat | Repeat | p-value | Non-Super-Utilizers | Super-Utilizers | p-value |
| --- | --- | --- | --- | --- | --- | --- |
| Sex *n* (%)          Male          Female | 35 (46.7)  40 (53.3) | 16 (32.7)  33 (67.3) | 0.121 | 41 (42.3)  56 (57.7) | 10 (37.0)  17 (63.0) | 0.625 |
| Race *n* (%)          White          Black          Other | 17 (22.7)  42 (56.0)  16 (21.3) | 9 (18.3)  36 (73.5)  4 (8.2) | 0.087 | 22 (22.7)  58 (59.8)  17 (17.5) | 4 (14.8)  20 (74.1)  3 (11.1) | 0.397 |
| Age (*mean ± SD* ) | 67.8 ± 17.6 | 67.2 ± 12.3 | 0.809 | 67.6 *±* 16.8 | 67.4 *±* 10.6 | 0.949 |
| Service Utilized *n (%)*          BLS          ALS | 1 (1.3)  74 (98.7) | 1 (2.0)  48 (98.0) | 1.000 | 95 (97.9)  2 (2.1) | 27 (100)  0 | 1.000 |
| Dementia *n (%)*          Present          Not documented | 6 (8.6)  64 (91.4) | 1 (2.1)  47 (97.9) | 0.238 | 6 (6.6)  91 (93.4) | 1 (3.7)  26 (96.3) | 1.000 |
| Altered Mental Status *n (%)*          Present          Absent | 1 (1.3)  74 (98.7) | 2 (4.1)  47 (95.9) | 0.562 | 1 (1.0)  96 (99.0) | 1 (3.7)  26 (96.2) | 1.000 |
| Anticoagulation or Antiplatelet  *n (%)*          Present          Absent | 30 (42.9)  40 (57.1) | 29 (61.7)  18 (38.3) | 0.046 | 44 (48.9)  46 (51.1) | 15 (55.6)  12 (44.4) | 0.543 |
| Completed Vital Signs *n (%)*          Yes          No | 51 (68.0)  24 (32.0) | 36 (73.5)  13 (26.5) | 0.515 | 51 (73.9)  18 (26.1) | 18 (66.7)  9 (33.3) | 0.525 |
| Vital Signs (*mean ± SD* )          HR          SBP          DBP          O2 Sat          RR | 84.6 (11.9)  136.9 (18.9)  79.6 (11.3)  97.8 (1.35)  17.4 (1.6) | 84.8 (12.5)  141.4 (18.9)  80.9 (9.3)  97.4 (3.6)  16.9 (1.9) | 0.903  0.219  0.569  0.398  0.129 | 85.3 (12.1)  139.3(18.4)  80.1 (10. 9)  97.5 (2.8)  17.3 (1.7) | 82.5 (11.9)  136.9 (16.1)  80.2 (8.5)  98.1 (1.4)  16.5 (1.8) | 0.326  0.583  0.961  0.402  0.034 |
| Living Conditions  *n (%)*          Alone          With Others | 36 (57.1)  27 (41.9) | 19 (50.0)  19 (50.0) | 0.485 | 47 (57.3)  35 (42.7) | 8 (42.1)  11 (57.9) | 0.230 |
| Ambulatory Status  *n (%)*          Walking          Wheelchair/ Bedbound | 44 (71.0)  18 (29.0) | 16 (43.2)  21 (56.8) | 0.006 | 49 (63.6)  28 (36.4) | 11 (50.0)  11 (50.0) | 0.248 |
